# Redefining and expanding the sphere of influence of BRCA in breast and colorectal cancers and beyond

**DOI:** 10.18632/oncotarget.28164

**Published:** 2022-01-14

**Authors:** Emil Lou

**Affiliations:** ^1^Masonic Cancer Center and Division of Hematology, Oncology and Transplantation, University of Minnesota, Minneapolis, MN 55455, USA

**Keywords:** BRCA1, BRCA2, BRCA, cancer genetics, breast cancer

***Commentary on:** Leaf et al. Opposing effects of BRCA1 mRNA expression on patient survival in breast and colorectal cancer and variations among African American, Asian, and younger patients. Oncotarget. 2021; 12:1992–2005. https://doi.org/10.18632/oncotarget.28082. [PubMed]*

In this issue, Leaf, Carlsen, and El-Deiry tackle the question of how expression of BRCA at the transcriptional level affects prognosis in breast and colorectal cancers, and furthermore what additional factors play a role [1]. While evaluation of BRCA1 has been so closely associated with breast cancer (the initials stemming from the words BReast CAncer point to that directly!), and to some extent ovarian cancer as well [2], any crossover impact with colorectal cancer (CRC) has not been well-defined until now. Genetic alteration of BRCA genes has been the most commonly studied aspect with clinical impact; specifically, mutation in BRCA has been associated with improved response of such tumors to platinum-based cytotoxic chemotherapies [3], and to targeted therapies inhibiting Poly ADP-ribose polymerase (PARP) [4]. To date this has been a blanket association, assuming that all mutations in BRCA have functional equivalence, and in some cases at the tumoral level have the same extent of penetrance and equal negative effect on DNA repair. Furthermore, in light of emergence of the sub-field studying ‘BRCAness’, a state of similar downstream effects result from alteration of players involved in homologous recombination DNA repair pathways, including isocitrate dehydrogenase (IDH)-1 and -2 [5]; unraveling associations of BRCA expression across cancers will reverberate and inform that field, and vice-versa. What has been less clear is the impact of differential expression of BRCA mRNA, independent of mutation status. The current study’s assessment and stratification of BRCA by mRNA levels addresses this clinical relevance and another important issue: genomic assessment at the DNA level doesn’t always show us an accurate picture of what genetic alterations are being expressed, and thus have a measurable and clinically meaningful downstream effect. Transcriptomic assessment as measured by mRNA helps to provide a more accurate picture of genomic impact. In this study, low BRCA1 mRNA was evaluated as a surrogate for loss of function occurring due to low expression.

BRCA is not a gene often associated with colorectal cancer (CRC), but the current study sheds some new light on potential subsets of this disease. In the United States, nearly 150,000 people are diagnosed with CRC each year, with incidence rising and of increasing concern in the young-adult population (defined as before age 50) [6]. What are the determinant genetic and earlier age-related factors that drive or otherwise allow carcinogenesis and development of tumors in patients diagnosed with early-onset CRC? The answer remains to be unraveled. *KRAS* mutations are present in >40% of CRC overall, but this number is significantly higher (~55%) in patients with young adult/early onset forms of CRC [7]. This disparity has critical implications for the notion that early onset CRC has a different biologic imprint and thus clinical behavior than CRC that occurs in older adults. This is especially important in young-onset CRC because of the role of mutated RAS in the early stages of CRC carcinogenesis in young adults (30–50) proposed by Vogelstein three decades ago [8]. New therapies that target the biological mechanisms of metastatic CRC are urgently needed to improve outcomes among patients whose disease is chemoresistant. The findings represented by Leaf et al. revealing that low BRCA1 mRNA expression in CRC correlates with poor survival not only stand in contrast to the findings in breast cancer patients, but also may provide new insight to genetic underpinnings of CRC in general. Interestingly, when stratified by age, there was a higher frequency of BRCA1 mRNA-high levels in cases of early-onset CRC. Furthermore, the authors also show that lower mRNA levels in CRC also align with stage IV disease and higher frequency of the mucinous form of adenocarcinoma, which is a subset that has higher proportion of peritoneal metastasis, worse morbidity, and worse prognosis [9]. Whether low BRCA mRNA points to a major underlying alteration that drives specific aggressive subtypes of CRC is an intriguing niche that merits further research in the mucinous subtype. The sum total of findings of negative association of low BRCA1 mRNA levels, but potential positive association of high mRNA in many early-onset cases, remains to be reconciled, as many such cases present with advanced-stage disease. Whether BRCA1 ultimately is more prognostic than predictive in this case bears further thought and investigation.

The variable findings of BRCA mRNA expression in African-American (more mRNA low cases in breast, but more mRNA high in CRC) and Asian patients (more mRNA high cases in breast, and the opposite in CRC), and other discoveries in this report, provided some surprises that stand in contrast to long-held assumptions of prognosis by histologic subtype. The findings open a Pandora’s Box of sorts as these levels did not match up as predicted with expected overall survival trends. The answer is that the effects are likely multi-factorial, as the authors readily acknowledge in the text. Some clues may come from their results from the overlapping gene set, as low BRCA1 mRNA levels correlated with low expression of other genes implicated in DNA repair (the DNA Topoisomerase Iiα TOP2A) and ATAD5, depletion of which instigates genomic instability. The findings presented here are a starting point for identifying which factors are causative or associated with efficacy of chemotherapy and overall prognosis.

Finally, in this era of precision medicine and at the same time of growing recognition and academic study of health disparities, research studies that objectively evaluate genetic risk factors and provide groundwork for health policy that will improve the lives of all populations affected by these cancers are crucial. Studies like this one provide background for better molecular-driven and evidence-informed screening strategies, and tailored treatment methods that should include equitable access to therapeutic clinical trials. Overall, transcriptomic evaluation of BRCA as reported in this study sheds valuable light on the biology of BRCA whose differential expression acts as a potential driver varying between cancers at different anatomic sites, and different patient populations based on age and other variables. The same methodology can be leveraged in future studies to analyze BRCA mRNA in other cancers affected by BRCA1, such as ovarian carcinomas, and also forms of tumors harboring BRCA2 alterations, including pancreatic adenocarcinomas. Congratulations and thanks to the authors for opening the door to asking these and even more questions in this expanding and important corner of molecular oncology.

## References

[1] Leaf S , Carlsen L , El-Deiry WS . Opposing effects of BRCA1 mRNA expression on patient survival in breast and colorectal cancer and variations among African American, Asian, and younger patients. Oncotarget. 2021; 12:1992–2005. 10.18632/oncotarget.28082. 34611475 PMC8487727

[2] Neff RT , Senter L , Salani R . *BRCA* mutation in ovarian cancer: testing, implications and treatment considerations . Ther Adv Med Oncol. 2017; 9:519–31. 10.1177/1758834017714993. 28794804 PMC5524247

[3] Mylavarapu S , Das A , Roy M . Role of BRCA Mutations in the Modulation of Response to Platinum Therapy. Front Oncol. 2018; 8:16. 10.3389/fonc.2018.00016. 29459887 PMC5807680

[4] Schettini F , Giudici F , Bernocchi O , Sirico M , Corona SP , Giuliano M , Locci M , Paris I , Scambia G , De Placido S , Rescigno P , Prat A , Curigliano G , Generali D . Poly (ADP-ribose) polymerase inhibitors in solid tumours: Systematic review and meta-analysis. Eur J Cancer. 2021; 149:134–52. 10.1016/j.ejca.2021.02.035. 33862496

[5] Sule A , Van Doorn J , Sundaram RK , Ganesa S , Vasquez JC , Bindra RS . Targeting IDH1/2 mutant cancers with combinations of ATR and PARP inhibitors. NAR Cancer. 2021; 3:zcab018. 10.1093/narcan/zcab018. 34027408 PMC8127964

[6] Siegel RL , Fedewa SA , Anderson WF , Miller KD , Ma J , Rosenberg PS , Jemal A . Colorectal Cancer Incidence Patterns in the United States, 1974–2013. J Natl Cancer Inst. 2017; 109:djw322. 10.1093/jnci/djw322. 28376186 PMC6059239

[7] Watson R , Liu TC , Ruzinova MB . High frequency of KRAS mutation in early onset colorectal adenocarcinoma: implications for pathogenesis. Hum Pathol. 2016; 56:163–70. 10.1016/j.humpath.2016.06.010. 27346571

[8] Fearon ER , Vogelstein B . A genetic model for colorectal tumorigenesis. Cell. 1990; 61:759–67. 10.1016/0092-8674(90)90186-i. 2188735

[9] Kim CW , Cha JM , Kwak MS . Identification of Potential Biomarkers and Biological Pathways for Poor Clinical Outcome in Mucinous Colorectal Adenocarcinoma. Cancers (Basel). 2021; 13:3280. 10.3390/cancers13133280. 34208938 PMC8268122

